# Nitrate Paradigm Does Not Hold Up for Sugarcane

**DOI:** 10.1371/journal.pone.0019045

**Published:** 2011-04-28

**Authors:** Nicole Robinson, Richard Brackin, Kerry Vinall, Fiona Soper, Jirko Holst, Harshi Gamage, Chanyarat Paungfoo-Lonhienne, Heinz Rennenberg, Prakash Lakshmanan, Susanne Schmidt

**Affiliations:** 1 School of Agriculture and Food Science, The University of Queensland, St Lucia, Queensland, Australia; 2 Institute for Forest Botany and Tree Physiology, University of Freiburg, Freiburg, Germany; 3 King Saud University, Riyadh, Saudi Arabia; 4 BSES Ltd, Indooroopilly, Queensland, Australia; Iowa State University, United States of America

## Abstract

Modern agriculture is based on the notion that nitrate is the main source of nitrogen (N) for crops, but nitrate is also the most mobile form of N and easily lost from soil. Efficient acquisition of nitrate by crops is therefore a prerequisite for avoiding off-site N pollution. Sugarcane is considered the most suitable tropical crop for biofuel production, but surprisingly high N fertilizer applications in main producer countries raise doubt about the sustainability of production and are at odds with a carbon-based crop. Examining reasons for the inefficient use of N fertilizer, we hypothesized that sugarcane resembles other giant tropical grasses which inhibit the production of nitrate in soil and differ from related grain crops with a confirmed ability to use nitrate. The results of our study support the hypothesis that N-replete sugarcane and ancestral species in the Andropogoneae supertribe strongly prefer ammonium over nitrate. Sugarcane differs from grain crops, sorghum and maize, which acquired both N sources equally well, while giant grass, *Erianthus,* displayed an intermediate ability to use nitrate. We conclude that discrimination against nitrate and a low capacity to store nitrate in shoots prevents commercial sugarcane varieties from taking advantage of the high nitrate concentrations in fertilized soils in the first three months of the growing season, leaving nitrate vulnerable to loss. Our study addresses a major caveat of sugarcane production and affords a strong basis for improvement through breeding cultivars with enhanced capacity to use nitrate as well as through agronomic measures that reduce nitrification in soil.

## Introduction

Synthetic nitrogen (N) fertilizer has facilitated dramatic yield increases in modern agriculture but only 30–50% of the annually applied 109 Tg of fertilizer-N is used by crops [1], [2]. The resulting pollution of land, water and atmosphere with reactive N is a pressing global problem, and it is imperative to improve the efficiency of N fertilizer use in crop systems [3]. Tropical and subtropical crop systems present a particular challenge because extreme rainfall events and weathered soils promote rapid N loss. Sugarcane is a food crop of industrial scale grown on 24 million hectares in the tropics and subtropics, producing 1.6 billion tons of biomass annually and providing the largest contribution of any crop to global biofuel production [2], [4]. While sugarcane is ranked the most suitable biofuel feedstock from energy conversion and environmental impact perspectives [5], its sustainability is arguable. Nitrogen fertilizer recovery by sugarcane is comparatively low and ranges from 20 to 40% [6], [7], [8] with up to 65% of applied N-fertilizer lost from the sugarcane-soil system [9]. These losses occur via pathways that include nitrate leaching, ammonia volatilisation, and gaseous emissions through microbial conversion of ammonium and nitrate [10]–[13]. Recent analysis of N-fertilized sugarcane soils showed that emissions of nitrous oxide, a potent greenhouse gas, exceed current IPCC estimates several-fold [11], [14]. While the potential for N_2_-fixing endophytes to contribute to the N budget has been reported in Brazilian sugarcane [15], the ability for biological nitrogen fixation to supply a considerable proportion of crop N-demand has not been substantiated in other high-production systems [16], [17].

Here, we explored reasons for the low N use efficiency of commercial sugarcane crops that receive the high N-fertilizer application rates characteristic of many producer countries. We examined the validity of the long-held paradigm that nitrate is the main N source for most crops. Nitrate is 5 to 10-times more mobile in soils than alternative N sources ammonium and amino acids [18]. While in natural ecosystems nitrification rates can be low and plants access a range of organic and inorganic N sources [19], [20], nitrification rates are generally high in agricultural systems [21]. Grain crops such as maize and sorghum have a high capacity to acquire and store nitrate in root and shoot tissues, a trait that has been targeted for breeding N-use-efficient maize cultivars [22]. We hypothesized that sugarcane has a preference for ammonium and a low capacity to use nitrate during periods of high N availability, and that discrimination against nitrate contributes to the pronounced accumulation of nitrate in sugarcane soils and subsequent N losses. In the context of global sugarcane production, we examined this hypothesis using several levels of experimental control.

## Results and Discussion

### Nitrogen fertilizer use is increasing in global sugarcane production

Approximately 2.5 Tg of fertilizer-N is applied in sugarcane production annually, accounting for 2% of global fertilizer-N use (Table 1). National averages of N harvested in millable stalks and removed from fields *versus* applied fertilizer-N indicate that N-efficiencies vary considerably across countries. More N-efficient countries, including Brazil and Philippines, have N removal/N-fertilizer input ratios of >0.6 while less efficient countries, including China and Pakistan, have ratios of 0.1 and 0.2 (Table 1). Average N-fertilizer application rates in Australia have declined in the past decade from 200 to 160 kg N ha^−1^ y^−1^ [23], but N applications are increasing elsewhere, and extreme rates of 400 to 750 kg N ha^−1^ y^−1^ occur in India and China (Table 1). Further, it is predicted that in Brazil alone the sugarcane producing area will double in the next decade to 14 million hectares [24], and N-fertilizer application rates are expected to increase as production expands to poorer soils [25]. The increasing N fertilizer applications in sugarcane crop systems contrast its status as the bioenergy crop with the most favourable N profile, which is based on the comparatively low use of N fertilizer in Brazilian sugarcane production [5]. Brazil currently accounts for 42% of global sugarcane production but only 25% of N-fertilizer use (Table 1). Our global analysis contradicts the general assessment; for example 2^nd^ and 3^rd^ ranked producer countries India and China produce 31% of global sugarcane but apply 50% of N-fertilizer (Table 1). Here we focus on Australian sugarcane systems which are characterized by high yields and an intermediate N harvest/N input ratio of 0.3.

**Table 1:** Sugarcane production and N fertilizer application of the top 14 sugarcane producing countries, which account for 86 and 88% of global sugarcane production area and cane yield, respectively.

Country	Area^a^(ha)	Production^a^(Tg)	N applied^b^(Gg)	N Removal^c^(Gg)	Output Input Ratio	Reported N application rates (kg N ha^−1^)
1. Brazil	8 141 135	649	613	389	0.6	60–100^1^
2. India	5 055 200	348	732	209	0.3	150–400^2^
3. China	1 708 520	125	512	75	0.1	100–755^3^
4. Thailand	1 054 439	74	44	44	1.0	
5. Pakistan	1 241 300	64	231	38	0.2	120–180^4^
6. Mexico	669 231	51	70	30	0.4	
7. Colombia	383 388	39	n/a	23		
8. Australia	390 000	34	70	20	0.3	160^5^
9. Argentina	355 000	30	25	18	0.7	
10. USA	374 200	28	40*	17	0.4	78–146^6^, 247–280^7^
11. Philippines	397 991	27	11	16	1.4	
12. Indonesia	415 578	26	52	16	0.3	125^4^
13. Guatemala	287 000	25	n/a	15		
14. South Africa	425 000	21	57	12	0.2	60–200^4^
**Total**	**20 897982**	**1 541**	**2 457**	**922**	**0.47** †	

a FAO area and yield data 2008.

b Heffer 2009 International Fertilizer Industry Association Assessment of fertilizer use by sugar crops at the global level 2007/2008.

*Figures for USA calculated from average application rate and area, sugarbeet production in remaining countries is minor compared to sugarcane.

c Calculated based on a stalk dry matter content of 30% and N content of 0.2% dry weight.

1 Hartemink [43].

2 Dr. T.K. Srivstava, Indian Council of Agricultural Research, India (*pers. comm*. 2010).

3 Dr. Jiang Xiong Liao, Guanxi Sugarcane Research Institute, China (*pers. comm*. 2010).

4 FAO Fertilizer use by crop (2005).

5 Average application rate Wood et al [23].

6 Louisiana State University Agricultural Center, 'Fertilizer Recommendations-(2009).

7 R. Rice, Institute of Food and Agricultural Sciences, University of Florida, USA (*pers. comm.* 2010).

† Weighted average output/input ratio.

### Nitrogen supply exceeds crop nitrogen demand in the first three months of the crop season

**Figure 1:**
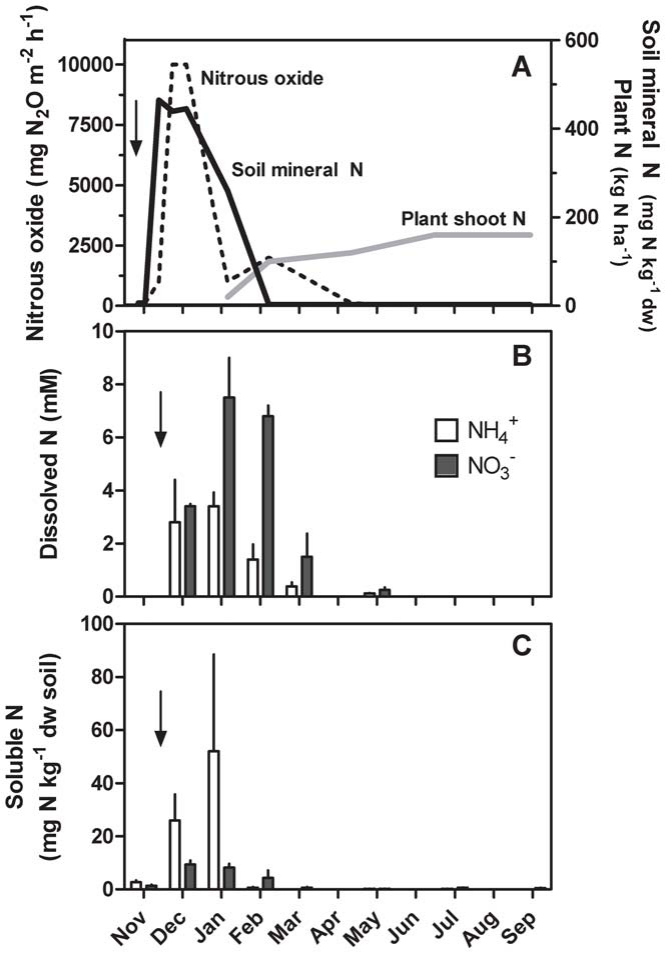
N availability throughout the crop cycle. (*A*) Soil nitrous oxide flux, soil mineral N (combined NH_4_^+^ and NO_3_^−^), and plant shoot N in the 3^rd^ ratoon of a sugarcane crop fertilised with urea at 200 kg N ha^−1^ as indicated by the arrow (redrawn from 6 and 11). Dissolved (*B*) and soluble (*C*) soil N over the growing cycle of a plant crop fertilised with urea at 110 kg N ha^−1^ as indicated by the arrow. (*B*) Dissolved NO_3_^-^ and NH_4_^+^ was measured in soil solution from 0–20 cm depth, and (*C*) soluble NO_3_^−^ and NH_4_^+^ measured in KCl extracts (0–20 cm), data represent mean±standard error n = 5 and n = 3, respectively.

Rapid growth and high rainfall in many sugarcane-growing regions prevent mechanical access to fields soon after crop establishment. Nitrogen fertilizer is therefore commonly supplied as a single application, resulting in high N concentrations in the soil. In most production areas sugarcane grows for 9–15 months and acquisition of nearly half of the final N in the crop can occur from three months post-fertilizer application in growing conditions typical for Australian sugarcane systems (Figure 1A, 6). The high availability of soil mineral N (nitrate and ammonium) early in the crop season is arguably poorly synchronised with plant N demand. The excessive N status of soil early in the crop season is further evidenced by the peak in nitrous oxide emissions (Figure 1A, 11).

We investigated the composition of the soil N pool because it has consequences for N acquisition and provides the basis for physiological experiments assessing N source preferences of sugarcane and related species. Concentrations of ammonium and nitrate in the soil solution (dissolved N pool) were similar soon after application of 110 kg N ha^−1^ (Figure 1B). In the following two months, ammonium concentrations remained at 1–3.5 mM while nitrate concentrations increased to 8 mM (Figure 1B). In contrast, the soluble N pool, which encompasses N bound to ion exchange sites, was dominated by ammonium during the first two months after fertilizer addition (Figure 1C). The increasing nitrate concentrations in the dissolved N pool are a likely result of nitrification rates exceeding nitrate acquisition rates of plants and microbes. The similar concentration of ammonium and nitrate in the dissolved N pool and prevalence of ammonium in the soluble pool soon after fertilizer application, together with previous studies of sugarcane soils [26], [27], contradict the generalisation that nitrate is the main N form in agricultural soils [28]. This highlights the need to consider the possibility that ammonium exerts negative feedback on nitrate uptake by sugarcane. We therefore explored whether ammonium-induced inhibition of nitrate uptake could contribute to the observed accumulation of nitrate in soil and N losses from sugarcane crop systems.

### Nitrogen-replete sugarcane preferentially acquires ammonium and discriminates against nitrate

The inherent difficulties associated with quantifying N turnover and interactions between plants, microbes and soil limit our knowledge of which N sources are used by soil-grown plants [29]. We therefore chose a suite of experimental approaches that spanned from inert-substrate and soil cultures in controlled glasshouse conditions to field-grown sugarcane for assessment of nitrate and ammonium uptake when supplied simultaneously.

**Figure 2:**
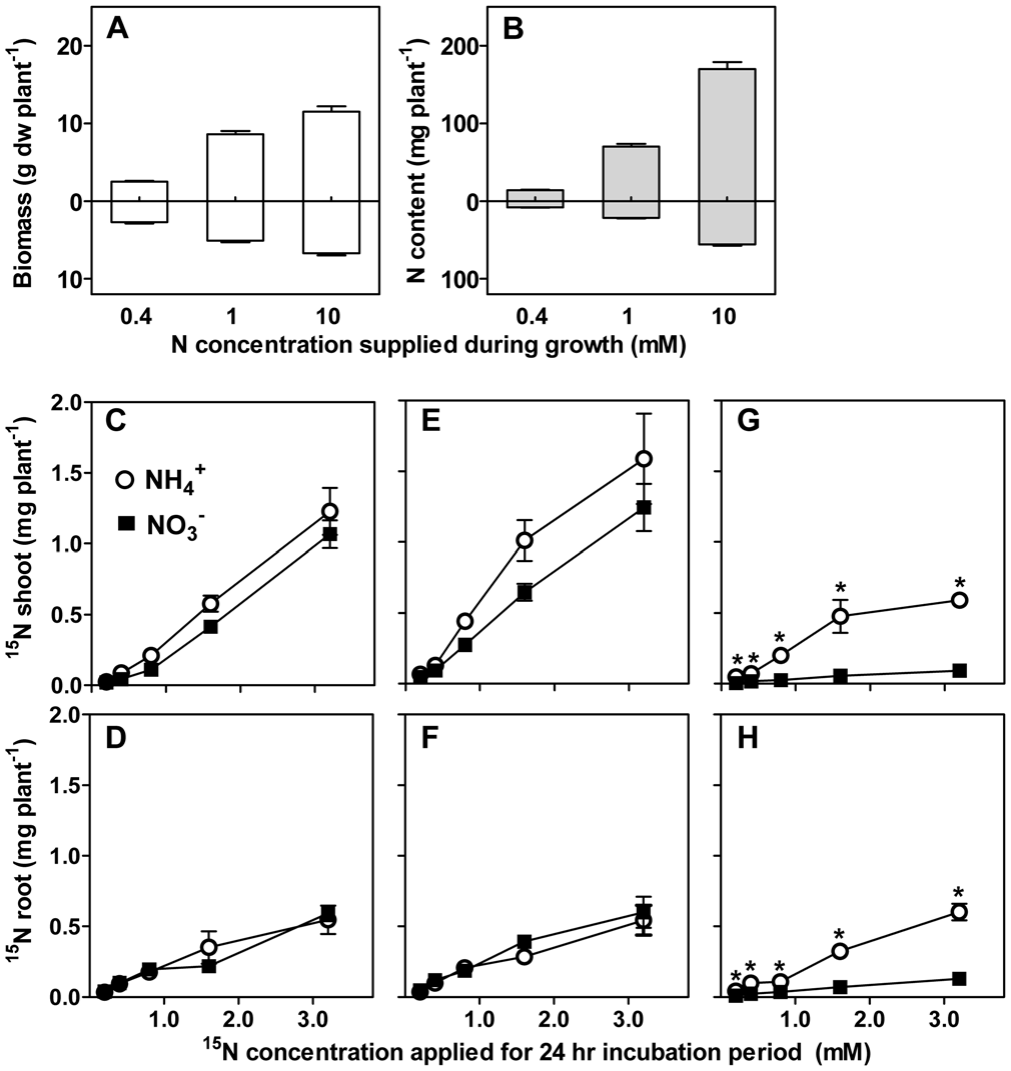
Nitrate and ammonium uptake with increasing N supply. Dry biomass (*A*) and total plant N content (*B*) for shoots and roots of sugarcane plants used for ^15^N labelling experiment with increasing N supply rates. Nitrogen treatments correspond to low (0.4 mM), intermediate (1 mM) and high (10 mM) N supply. Bars represent mean±standard error (n = 20). Data from three commercial sugarcane varieties were pooled. Uptake of ^15^N into shoots (C, *E*, *G*) and roots (*D*, *F*, *H*) of plants grown at low (0.4 mM N) (*C, D*), intermediate (1 mM N) (*E, F*) and high (10 mM N) (*G, H*) N supply. ^15^N was supplied to plants as either ^15^NH_4_NO_3_ (○) or NH_4_^15^NO_3_ (▪) at concentrations from 0.2 to 3.2 mM ^15^N for 24 h. Data represent mean±standard error (n = 3). * indicate significance difference between NH_4_ and NO_3_ uptake at *P*<0.05 (ANOVA on ln transformed data, Tukey's post hoc test).

Considering the potential effect of plant N status on N source preferences, low-to-high concentrations of ^15^NH_4_NO_3_ or NH_4_^15^NO_3_ were supplied over 24 h to plants grown at limiting (0.4 mM N), intermediate (1 mM N) and high (10 mM N, ‘N-replete’) N supply in inert substrate. Biomass and N content increased significantly (*P*<0.001) with N supply rate; low and intermediate N-grown plants produced 26 and 76% of the biomass of N-replete plants (Figure 2A), while N content of low and intermediate N-grown plants was 12 and 40% of N-replete plants (Figure 2B). Low and intermediate N-grown plants incorporated equal amounts of ^15^N-nitrate and ^15^N-ammonium into shoots and roots (Figure 2C-F). In contrast, N-replete plants incorporated significantly (*P*<0.05) less ^15^N-nitrate than ^15^N-ammonium. Incorporation of ^15^N-nitrate was 5-times lower in roots and 4 to 10-times lower in shoots than ^15^N-ammonium incorporation (Figure 2G, H). No nitrate was detected in shoots or in roots of low or intermediate N supplied plants indicative of rapid nitrate assimilation, while roots of N-replete plants contained 10–15 µmol NO_3_^−^ g^−1^ dw at all ^15^N-labelling rates.

The observed discrimination against ^15^N-nitrate in N-replete plants is consistent with other crops which reduce nitrate uptake in the presence of ammonium in hydroponic conditions [30], but this has not been considered in sugarcane production. The high availability of dissolved and soluble ammonium in sugarcane soil early in the crop season together with the observation that N-replete sugarcane discriminates strongly against nitrate in the presence of both N sources, suggests that nitrate is not acquired as efficiently as ammonium. To examine this further, we studied early-season sugarcane in a field setting.

### Uptake of ammonium exceeds nitrate in roots of field-grown sugarcane

**Figure 3:**
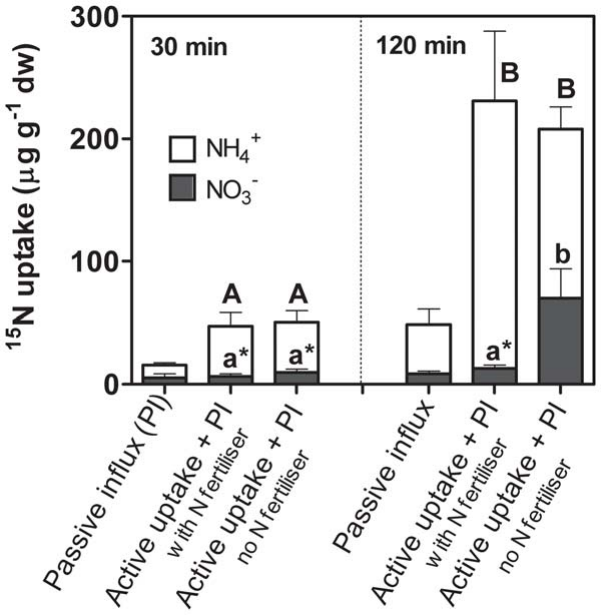
Nitrate and ammonium uptake into intact roots. ^15^N incorporation into attached, freshly excavated roots of fertilized (+140 kg urea N ha^−1^) and unfertilized field grown 3-month-old plants when supplied with 1 mM N NH_4_^15^NO_3_ (▪) or ^15^NH_4_NO_3_ (□) for 30 and 120 min. Roots treated with protonophore, CCCP, prior to incubation indicate passive influx (PI). Bars represent mean±standard error (n = 4). ^A,a^ indicate significance difference between fertilized and unfertilized uptake for NH_4_^+^ and NO_3_^−^; * indicate significance difference between NH_4_^+^ and NO_3_^−^ uptake at *P*<0.05 (ANOVA, Tukey's post hoc test).

Nitrogen uptake by sugarcane roots in a commercial field early in the crop cycle confirmed that N-replete plants discriminate against nitrate. Two weeks prior to the experiment, half of the test plants received a commercial N-fertilizer rate, and the other half remained unfertilized. Equimolar concentrations of ^15^NH_4_NO_3_ or NH_4_^15^NO_3_ were supplied to carefully excavated roots that remained attached to plants. Leaf transpiration rates remained steady over the duration of the experiment indicating that root excavation did not perturb plant function. Passive influx of ^15^N, not requiring ATP, accounted for 33% and 21% of total ^15^N incorporation after incubation for 30 and 120 minutes, respectively (Figure 3). ^15^N-ammonium incorporation was significantly (*P*<0.05) higher than ^15^N-nitrate incorporation in roots of unfertilized and fertilized plants after 30 minutes (Figure 3). ^15^N-nitrate incorporation was 15% of ^15^N-ammonium incorporation in roots after 30 minutes incubation and did not increase in roots of fertilized plants after 120 minutes incubation. In unfertilized plants, ^15^N-nitrate incorporation increased with incubation time and was ∼6-fold higher than in roots of fertilized plants after 120 minutes (Figure 3).

Prior to the incubation, roots of fertilized plants contained ∼3-fold more nitrate than unfertilized plants (28 and 8 µmol NO_3_^−^ g^−1^ dry weight, respectively) while nitrate contents of shoots were similar (11 and 9 µmol NO_3_^−^ g^−1^ dry weight). Nitrogen content of roots and shoots of unfertilized and fertilized plants were similar indicating that differences in N status are only evident in the higher nitrate content of roots of fertilized plants. Increasing nitrate incorporation of unfertilized sugarcane over the 120 minutes incubation period suggests that nitrate transporters were induced in root membranes facilitating transport of nitrate into root cells. Similarly, supply of nitrate to roots of Arabidopsis increased mRNA levels of a dual-affinity nitrate transporter, with K_m_ values of 50 and 4000 µM for high and low-affinity phases, after 30 minutes and nitrate uptake increased in the following 2 to 3 hours [31]. In the N-replete plants studied here, there is little evidence of nitrate influx via low affinity transporters, which are active in the higher N concentration range used in our experiment (500 µM) and considered less sensitive to feedback mechanisms [28].

We conclude that induction of nitrate transporters in unfertilized plants is a likely response to the nitrate supplied in the incubation solution, and supports our previous results that only N-limited plants acquire nitrate at a similar rate as ammonium. In the following experiments, we compared N source preferences and nitrate storage of sugarcane with those of related ancestral and grain crop species in controlled glasshouse conditions.

### Commercial sugarcane and ancestral *Saccharum* species have lower uptake and storage of nitrate than related giant grass and grain crop species

To provide insight into nitrate use and the regulatory mechanisms involved, nitrate storage and uptake of ammonium and nitrate were assessed in closely related grass species in two experimental comparisons. In the first comparison, 3 genotypes each of the two ancestral species of commercial sugarcane, *Saccharum spontaneum* and *S. officinarum*, as well as *Saccharum-Erianthus* hybrids and *Erianthus* species were grown in inert substrate and well supplied with N. The N-replete plants received equimolar ^15^N/^14^N/ammonium/nitrate/glycine solutions for 24 hours. Glycine was added because amino acids account for a considerable proportion of the soluble N pool of sugarcane soil later in the growing season, but this was not the focus here. In the second comparison, commercial sugarcane, *S. spontaneum, Erianthus arundinaceus*, sorghum and maize were grown in soil characterized by a low ion exchange capacity and well supplied with N. Uptake of nitrate and ammonium was assessed with ^15^N/^14^N/ammonium/nitrate solutions over a shorter period (2 h) to minimise the effect of microbial conversion of the supplied N.

**Figure 4:**
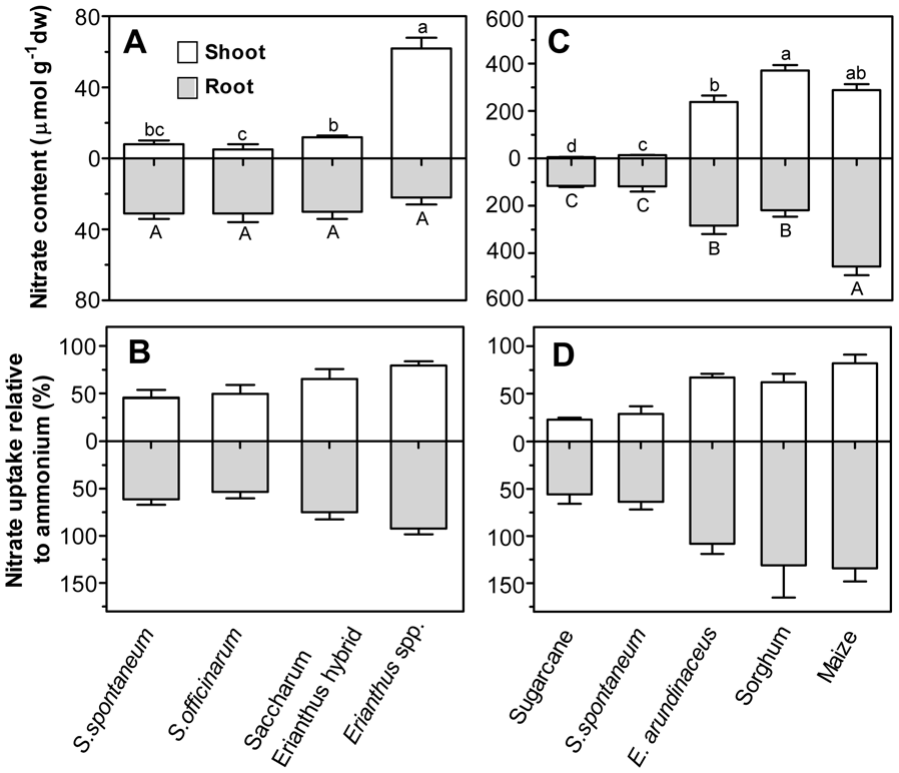
Nitrate use across Andropogoneae supertribe. (*A*) Shoot and root nitrate content (µmol g^−1^ dw) of *Saccharum spontaneum* cultivars, *S. officinarum* cultivars, *Saccharum-Erianthus* hybrids and *Erianthus* species grown for 12 weeks in perlite growth medium with adequate N supplied as equimolar NH_4_^+^, NO_3_^−^, Gly and (*B*) relative ^15^N content of shoot and root of the same plants supplied with NH_4_^15^NO_3_ Gly compared to those supplied with ^15^NH_4_NO_3_Gly for 24 h. Values for 3 cultivars (n = 4) were pooled for each group. (*C*) Shoot and root nitrate content (µmol g^−1^ dw) of sugarcane, *S. spontaneum, Erianthus arundinaceus*, sorghum and maize grown for 5 weeks in soil with adequate N supplied as equimolar NH_4_NO_3_ and (*D*) relative ^15^N content of shoot and root of the same plants supplied with NH_4_^15^NO_3_ compared to those supplied with ^15^NH_4_NO_3_ for 2 h. (*A, C*) Bars represent averages±standard error (n = 8). ^A,a^ indicate significance difference between genotypes *P*<0.05 (ANOVA, Tukey's post hoc test). (*B, D)* Values are the ratio averaged from comparisons within each of the 4 replicates, with standard error.

In both comparisons, sugarcane and *Saccharum* species demonstrated a low capacity to store nitrate in shoots as evidenced by low concentrations of 5 to 13 µmol nitrate gdw^−1^ (Figure 4A, C). In inert substrate, nitrate concentration of shoots was 8-fold greater in *Erianthus* species than *Saccharum* species and *Saccharum-Erianthus* hybrids (Figure 4A). In soil-grown plants, shoot nitrate concentrations were 20 to 37-fold higher in maize, sorghum and *E. arundinaceus* than in sugarcane and *S. spontaneum* (Figure 4C). The different nitrate accumulation detected in our experiments resembles field-grown *Erianthus* and sorghum which had 8 to 30-fold higher nitrate concentrations in stems than sugarcane [32]. In contrast to shoots, root nitrate concentrations of *Saccharum* species were similar to those of *Erianthus* species grown in inert culture (Figure 4A), while maximum root concentrations of soil-grown sugarcane and *S. spontaneum* were 100 µmol nitrate gdw^−1^, approximately 2- to 4-fold lower than the other species (Figure 4C).

Overall, nitrate was a minor contributor to the total N pool in *Saccharum* species, including sugarcane, accounting for 2 to 5% compared to 22% in maize when calculated on a whole plant basis (Figure 4, Table 2). This contrast was even greater when considering only aboveground tissues as nitrate comprised 0.5–0.8% and 17% of the total N pool in *Saccharum* species and maize, respectively (Figure 4, Table 2). Similarly, nitrate content in field grown wheat comprised up to 18% of the total N of aboveground tissue [33]. Stored nitrate in plant tissues demonstrates a capacity to acquire N in excess of assimilation rates and the critical content of reduced N required for maximal growth. Commercial sugarcane had higher biomass and lower tissue N concentrations than the other species indicating a greater ability of sugarcane to generate biomass per unit tissue N (Table 2). However, total N uptake per plant was similar in all species with the exception of a lower N uptake by *S. spontaneum*. The lack of nitrate storage in shoots of *Saccharum* species may indicate rapid assimilation of nitrate in shoots and/or low uptake of nitrate, and we investigated these possibilities by quantifying the incorporation of ^15^N-nitrate into tissues.

**Table 2:** Biomass, N content, transpiration and ^15^N tissue concentration and recovery of sugarcane and related species after 5 weeks growth with adequate N supply.

	Sugarcane	*Saccharum spontaneum*	*Erianthus arundinaceus*	Sorghum	Maize
**Biomass (g)**					
Shoot	13.5^a^	7.4^b^	7.6^b^	7.2^b^	9.8^b^
Root	8.9^a^	2.4^c^	3.9^bc^	5.2^b^	4.8^bc^
**N content (mg N dw^−1^)**					
Shoot	16.5^d^	21.2^c^	26.4^b^	31.9^a^	23.6^bc^
Root	9.8^c^	13.5^b^	13.8^b^	14.2^b^	17.0^a^
**Transpiration**					
**(mmol m^−2^ s^−1^)**	299^ab^	193^b^	401^a^	352^a^	335^ab^
**Leaf Area (cm^2^)**	930^c^	491^d^	819^c^	1244^b^	2029^a^
**^15^N content (µg g^−1^ dw)**					
^15^NH_4_^+^ supplied					
Shoot	86^C^	81^C^	110^BC^	195^A^	154^AB^
Root	257^A^	140^C^	167^BC^	241^AB^	318^A^
Recovery of ^15^NH_4_^+^ (%)	12.3	2.9	5.4	7.6	11.1
^15^NO_3_^−^ supplied					
Shoot	20^B^ *	26^B^ *	74^A^	120^A^	126^A^
Root	143^CD^ *	90^D^	190^BC^	304^AB^	427^A^
Recovery of ^15^NO_3_^−^ (%)	4.5	1.4	3.2	8.8	9.6

Nitrate content and relative ^15^N incorporation of these plants is shown in Figure 4 C, D. ANOVA and Tukeys post hoc test (*P*<0.05) were performed on ln-transformed data.

a indicates significant differences in biomass, transpiration and N content between genotypes.

A indicates significant differences in ^15^N concentration between genotypes supplied with ^15^NH_4_NO_3_, and between genotypes supplied with NH_4_^15^NO_3_ for each tissue type.

*indicate significantly lower ^15^N concentration within genotype when supplied with NH_4_^15^NO_3_ compared to ^15^NH_4_NO_3_. Data represent mean n = 8 for biomass, N content data and n = 4 for ^15^N data.

In inert substrate culture, *S. spontaneum* and *S. officinarum* had a lower relative ^15^N-nitrate uptake than *Erianthus arundinaceus* and *Saccharum*-*Erianthus* hybrids (Figure 4B). ^15^N-nitrate uptake into shoots was 46–50% of ammonium uptake in *Saccharum* spp. compared to 80% in *Erianthus* spp. (Figure 4B). Similarly in soil culture, sugarcane and *S. spontaneum* incorporated significantly less nitrate than *E. arundinaceus*, sorghum and maize (Figure 4D), and accumulation of ^15^N-nitrate was significantly lower (*P*<0.05) in roots and shoots of sugarcane and in shoots of *S. spontaneum* than ^15^N-ammonium (Table 2). Recovery of ^15^N applied as nitrate and ammonium in sugarcane was 4.5 and 12.3%, compared to 9.6 and 11.1% in maize and 8.8 and 7.6% in sorghum, indicating that the low nitrate uptake by sugarcane is not compensated for with an increased incorporation of ammonium (Table 2).

The leaf area of maize was ∼2-fold higher than that of sugarcane (Table 2). However, the similarity of transpiration rates together with the lack of correlation leaf area and ^15^N-nitrate uptake within and across all species suggest that the differences in nitrate uptake cannot be attributed solely to mass flow driving nitrate delivery to the root surface [34].

The low nitrate content in shoots of commercial sugarcane cultivars and *Saccharum* species together with low translocation rates of ^15^N-nitrate to shoots suggest a bottleneck in the uptake and root-to-shoot transport of nitrate rather than limitation in nitrate assimilation *per se*. Negative feedback from endogenous nitrate on transport systems can be inferred from whole plant or organ studies that show negative correlation between nitrate concentration and uptake rate [35], although nitrate or assimilatory products ammonium and amino acids act as regulatory signals. For example, nitrate concentrations in the cytosol of barley roots remained at 4 mM whereas vacuolar nitrate concentrations increased from 4 to 75 mM when plants were supplied with 0.01 to 10 mM nitrate [36]. Our results presented here indicate that nitrate uptake is inhibited in N-replete sugarcane and that this is correlated with increasing nitrate content in roots.

The advantage of nitrate as a N source is the uncoupling of N supply and demand, while ammonium causes toxicity in cells which necessitates rapid assimilation and is limited by the carbon supply to roots [37]. Many nitrophile species exhibit efficient use of nitrate through rapid transport and storage of nitrate which is considered an evolutionary advanced character in angiosperms [38]. Thus, N uptake in excess of demand and the resulting storage of nitrate occurs when excess ammonium and nitrate are supplied to sorghum and maize but not sugarcane and related species. The intermediate status of *Erianthus arundinaceus* is suggestive of a continuum of nitrate use in the Andropogoneae supertribe. Understanding the mechanisms involved in nitrate use of sugarcane including root uptake, root-to-shoot transport, storage, remobilisation and assimilation is now required. Together the findings suggest a broader spectrum of N use among the studied crop and wild species than previously recognised and question the assumed efficient use of nitrate in sugarcane crop systems.

As demand for sugarcane as an agricultural and biofuel commodity rises, sustainable N use is a key prerequisite. We show that sugarcane differs from grain crops in the ability to take up and store nitrate. Rather sugarcane resembles related tropical grasses in the Andropogoneae supertribe, such as *Andropogon gayanus* which has a low capacity for nitrate uptake and inhibits microbial nitrification in soil [39]–[41]. The preference of sugarcane for ammonium and concomitant discrimination against nitrate in well N-supplied growth conditions characteristic of the first three months of the crop season exacerbates the disparity between N supply and plant N use. The superior ability of *Erianthus arundinaceus* to acquire and store nitrate may positively influence sugarcane breeding programmes aimed at improved nitrate use of sugarcane. Improved cropping system and fertilizer management aimed to reducing nitrate content in soils in favour of ammonium and organic N forms should also be a priority.

## Materials and Methods

### Availability of dissolved and soluble soil N throughout the crop cycle

Soil mineral N was sampled from a Q117 plant crop at a subtropical site near Bundaberg Queensland, Australia. The soil was characterized as a yellow to brown dermosol with the top 20 cm a sandy loam. The site was fertilized with ammonium-urea at 110 kg N ha^−1^ on 12 Dec 2008. Soil was sampled 8 times (once prior to fertilizer addition in Nov 2008, then subsequently in Dec 2008, Jan, Feb, Mar, May, Jul and Sept 2009). The top 20 cm of soil was collected with an auger, kept at stable temperature then analyzed within 24 h. Soil solution containing the dissolved N fraction was collected from approximately 25 g of fresh soil by centrifugation for 45 min at 4500 rpm. Soluble N was extracted using 1.5 M KCl in a 1∶2 soil:solution ratio, shaken for 1 h then centrifuged at 4000 rpm for 10 min. Supernatant containing soluble N and soil solutions containing dissolved N were analysed for nitrate by colorimetric assay [42] and ammonium by liquid chromatography using an UPLC (Ultra Pressure Liquid Chromatography Unit, Waters, Milford, USA), equipped with a BEH C_18_ 1.7µm 2.1×50 mm analytical column and a tuneable UV detector at 254 nm. Extracts were derivatized using the AccQ-Tag™ derivatisation kit (Waters, Milford, USA).

### Comparison of ammonium and nitrate uptake in N-limited and N-replete sugarcane with increasing concentrations

Commercial sugarcane cultivars, *Saccharum* spp. hybrids, Q138, Q157 and Q179^A^ were grown from setts (stem cuttings) in coarse perlite within 15 cm diameter free-draining pots (2 L), and kept in a naturally lit glasshouse for 3 months. Plants received 200 mL of nutrient solution (pH 5.6) containing 0.2, 0.5 or 5 mM NH_4_NO_3_ and other nutrients as; 1 mM K_2_SO_4_, 2 mM MgSO_4_, 2 mM CaSO_4_, 0.457 mM KH_2_PO_4_, 42.5 µM K_2_HPO_4_, 100 µM FeEDTA, 20 µM MnSO_4_, 10 µM H_3_BO_3_, 1 µM CuSO_4_, 2.5 µM ZnSO_4_, 0.35 µM Na_2_MoO_4_ and tap water on alternate days. CaSO_4_ was added to lower N treatments to maintain equivalent osmolarity. ^15^N-labelled ammonium or nitrate (98 atom% excess) were added to each plant at 0.2, 0.4, 0.8, 1.6 or 3.2 mM ^15^N with equimolar concentrations of the unlabelled alternative N form. After 24 h, pots were rinsed with 10 mM KCl and roots cleaned of perlite. Plant tissue was dried at 55°C for 3 days and analyzed for ^15^N content and nitrate content.

### Comparison of ammonium and nitrate uptake into roots of field grown commercial sugarcane

Attached roots of fertilized (140 kg urea N ha^−1^, 100 kg K ha^−1^ applied 2 weeks prior to sampling) and unfertilized (100 kg K ha^−1^) 3-month old KQ228^A^, were excavated approximately 20 cm from the plant base, kept moist and incubated *in situ* in buffered nutrient solution (pH 6.0, 0.025 mM KH_2_PO_4_, 0.025 mM K_2_HPO_4_, 0.125 mM K_2_SO_4_, 0.1 mM MgSO_4_, 0.2 mM CaSO_4_) with either 1 mM N of NH_4_^15^NO_3_ or ^15^NH_4_NO_3_ (98 atom%) for 30 and 120 min at 25°C. Roots of a subset of plants from the fertilized plots were pre-treated with 50 µM carbonyl cyanide 3-chlorophenylhydrazone (CCCP), a protonophore, 30 min prior to ^15^N addition to indicate passive uptake. Roots were removed from plants, rinsed in 10 mM KCl, then water and dried at 55°C. Separate plants were used for each ^15^N source, CCCP protonophore treatment and time point combination and all treatments were repeated on 4 consecutive days. Entire aboveground shoot and incubated root material were analyzed for ^15^N content. No ^15^N was detected in shoot tissue of any treatment.

### Comparison of nitrate use across *Andropogoneae* supertribe

Experiment 1: Twelve genotypes including 3*S. spontaneum* cultivars (IK 76–86, US 71-4-1, *S. spontaneum* hybrid CT04-275); 3*S. officinarum* cultivars (Manjri Red, Keong Java, NG 57–239); 3 *Saccharum-Erianthus* hybrids of 3^rd^ generation [Badilla*Erianthus]* CP84-1198]*ROC20, (CT06-381, CT06-389, CT06-376); and 3 *Erianthus* spp. (*Erianthus procerus* SES 309, *Erianthus arundinaceus* IK 76–63, IJ 76–394) were grown in perlite medium within 2 L, 15 cm diameter pots for 3 months with high N supply (equimolar ammonium, nitrate and glycine). N supply increased from 3 mM N at the start of the experiment to a final concentration of 30 mM N to ensure that plants were well supplied with N. Other nutrients were supplied as 5 mM K_2_SO_4_, 2 mM MgSO_4_, 2 mM CaSO_4_, 0.457 mM KH_2_PO_4_, 42.5 µM K_2_HPO_4_, 100 µM FeEDTA, 20 µM MnSO_4_, 10 µM H_3_BO_3_, 1 µM CuSO_4_, 2.5 µM ZnSO_4_, 0.35 µM Na_2_MoO_4_ with tap water supplied on alternate days. To compare uptake of different N forms 200 ml of solutions containing ^15^N-labelled ammonium, glycine or nitrate (98 atom%) were added to each plant with the equimolar concentration of the alternative N forms supplied as unlabelled ammonium, glycine and nitrate. After 24 h, pots were thoroughly rinsed with 10 mM KCl prior to harvest. Roots were carefully removed from pots, cleaned of perlite, rinsed with water, then dried and prepared for ^15^N analysis. Data from the 3 cultivars within each group were pooled for statistical analysis. *Saccharum-Erianthus* hybrids were generated by the Guangzhou Sugarcane Industry Research Institute at the Hainan Sugarcane Breeding Station, Hainan province, China. *S. spontaneum* and *S. officinarum* cultivars were supplied by BSES, Meringa, Australia.

Experiment 2: *Zea mays* (Hycorn 624, supplied by Pacific Seeds, Toowoomba, Australia), *Sorghum bicolour* (A35/RQL36, supplied by DEEDI Hermitage Research Station, Warwick, Australia), *Erianthus arundinaceus* (IJ76–394), *Saccharum spontaneum* (US71-4-1) and commercial sugarcane cultivar *Saccharum* hybrid (Q232^A^) were germinated and grown in a sandy loam (pH 4.7, EC 200 µScm^−1^, %C 0.4, %N 0.002) within 15 cm diameter free-draining pots (10 L) for 5 weeks in a glasshouse. Plants were supplied with 200 mL 10 mM NH_4_NO_3_ and other nutrients, as listed in experiment 1, 3 times a week and supplemented with tap water throughout. In the week preceding ^15^N application and harvest, stomatal conductance was measured on the youngest fully expanded leaf using a leaf porometer (Decagon Devices, Washington, USA) over 3 days between 8am and 1pm. Plants were supplied with either 10 mM ^15^NH_4_NO_3_ or NH_4_^15^NO_3_ (98 atom% excess) for 2 h from 9 to 11 am, after which the above-ground shoots of all plants were immediately removed, separated into leaf blade and stalk and dried. Prior to drying the surface area of leaf blades was measured for each plant with a Licor LI-3100C (Licor, Nebraska, USA). Roots were removed from soil, rinsed in 10 mM KCl, then water and dried at 55°C for 3 days. Each of the 4 replicates was harvested on consecutive days. All species were in their vegetative growth stage to avoid phenology-based differences in N relations.

### Plant tissue analysis

Dried shoot and root material from glasshouse and field experiments were ground to a fine powder (Retsch ball mill, MM-2, Haan, Germany) and analyzed for ^15^N and %N using an Integra-CN (Sercon Ltd., Cheshire, UK) at the UC Davis Stable Isotope Facility (Davis, California, USA). Nitrate content was determined on 20% methanol dried tissue extracts [42].

### Statistical analysis

Data were analyzed using Statistica 9.0 (StatSoft Inc., Tulsa, Oklahoma, USA). Significant differences between treatments were determined using (ANOVA) P<0.05 followed by Tukey's *post hoc* test and data transformed where indicated throughout text.
